# Driving and cannabis use: a questionnaire about knowledge and behaviors after the legalization of recreational cannabis in California

**DOI:** 10.1186/s12889-025-24309-4

**Published:** 2025-09-30

**Authors:** Sara Baird, Daniel Ageze, Linda L. Hill, Sarah Hacker, Renee Dell’Acqua, Alice Gold, Ilene Lanin-Kettering, Tom Shaughnessy, Thomas D. Marcotte

**Affiliations:** 1 Herbert Wertheim School of Public Health and Longevity Science, University of California San Diego, 9500 Gilman Drive, La Jolla, CA 92093 USA; 2 Center for Medicinal Cannabis Research, Department of Psychiatry, University of California San Diego, 220 Dickinson Street, Suite B, San Diego, CA 92103 USA; 3 Quester, 6500 University Avenue Suite 205, Des Moines, IA 50324 USA

**Keywords:** Cannabis, Driving under the influence of cannabis (DUIC), Driving under the influence (DUI), Marijuana, Driving, Proposition 64

## Abstract

**Background:**

Implemented in 2018, Proposition 64: The Adult Use of Marijuana Act legalized recreational cannabis use in California. This study aimed to assess driving-related knowledge, attitudes, and behaviors after the passage of Proposition 64.

**Methods:**

An initial questionnaire was completed by 15,208 participants demographically matched to the 2020 California census. A subset of 4,020 participants who currently use cannabis, 523 who formerly used cannabis, and 635 who never used cannabis completed a detailed mixed qualitative and quantitative questionnaire, including questions about driving which were selected for this sub-analysis. Chi-square analysis was utilized for descriptive analysis. For this study “cannabis” was defined as THC-containing products.

**Results:**

62% of current cannabis users were aware that drivers and passengers cannot smoke or ingest cannabis in a moving vehicle, and 59% were aware that any container of cannabis inside a moving vehicle must be unopened and sealed. 74% knew that you could get a citation for driving under the influence of cannabis (DUIC). 64% of participants reported feeling safe to drive 3 h or less after inhalation of flower products and 55 % felt safe to drive 5 h or less after consumption of edible cannabis products. 13% reported that the passage of Proposition 64 increased their Likelihood of DUIC. Those with lower knowledge of Prop 64 driving related regulations were more likely to ever have been pulled over or involved in a crash while under the influence of cannabis.

**Conclusions:**

Six years after legalization implementation, there remains mixed awareness of driving-related regulations among people who currently use cannabis. Lower knowledge of regulation was associated with an increase in adverse driving outcomes. Effective messaging to increase knowledge of regulations, duration of intoxication, and promotion of safe driving behaviors is an essential step for promoting public safety after the legalization of cannabis.

**Trial registration:**

N/A

**Supplementary Information:**

The online version contains supplementary material available at 10.1186/s12889-025-24309-4.

## Background

As of 2024, cannabis surpassed alcohol as the impairing substance used most frequently on a daily or near daily basis among individuals 12 years of age and older in the United States [1]. In 2016, California voters passed ballot measure Proposition 64 (Prop 64), also known as the Adult Use of Marijuana Act. Implemented in 2018, Prop 64 legalized the recreational use of cannabis for adults in California aged 21 and older. In addition to legalizing the possession, use, and cultivation of limited amounts of cannabis for personal use, Prop 64 introduced taxation guidelines, outlined requirements for distribution and sale, and introduced restrictions related to cannabis and motor vehicles [2].

In general, driving-related restrictions of cannabis are similar to those related to the use of alcohol. Unlike with alcohol, though, where per se limits for breath alcohol concentrations (BAC) are well-established, there are no widely accepted cutpoints indicative of impairment for delta-9 tetrahydrocannabinol (THC) in blood given its complex pharmacokinetics and pharmacodynamics [3]. Under Prop 64, it is prohibited to smoke or ingest cannabis, or possess an open package of cannabis, while driving or riding in a motor vehicle. Driving under the influence of cannabis (DUIC) remains unlawful, as previously established by California Vehicle Code 23,152 [4]. Yet even before the legalization of recreational cannabis, cannabis was one of the most common illicit substances detected in motor vehicle operators driving under the influence [5]. A 2010 study found that 8.5% of California drivers tested during weekend nighttime hours were positive for THC [6]. California’s Roadside Survey of Alcohol and Drug Use in 2012 found a THC test positivity rate of 14.0% among nighttime weekend drivers [7]. However, there is a poor correlation between THC levels and degree of impairment, which depends on multiple factors such as the product used, route of administration, and personal tolerance, and roadside tests for assessing cannabis intoxication have limitations [3, 8–10]. In part, people who use cannabis may make their own determination as to whether they are too impaired to drive. In the 2018 National Survey on Drug Use and Health, 4.7% of US residents aged 16 and over reported driving a vehicle while under the influence of cannabis in the past 12 months [11].

Research shows conflicting data on how cannabis legalization impacts driving behavior and outcomes. A U.S. national cross-sectional sample of past-month cannabis users found that individuals in states without any cannabis legalization were more likely to self-report driving under the influence in the past month (40%), defined as driving within 3 h of “getting high,” than those in states with recreational (29%) or medical (27%) legalization laws [12]. Similarly, the post-legalization Canadian National Cannabis Survey found that 26% of past-year cannabis users drove within two hours of cannabis inhalation, with higher rates observed among men and individuals 25 years of age and older [13]. Studies in states and countries that have legalized adult cannabis use have reported increases in the detection of THC in drivers involved in traffic crashes and crash fatalities [14–17], although causality is difficult to determine.

There is a lack of research examining public knowledge of specific components of cannabis legalization laws. In the same U.S. survey of adults from states both with and without recreational legalization, perceived safety but not perceived legality was associated with an increased likelihood of DUIC [12]. Among frequent users, the presence of state cannabis driving laws was associated with a lower likelihood of DUIC [18].

In 2021, the Herbert Wertheim School of Public Health and Human Longevity Science at University of California San Diego (UC San Diego) began the Impact 64 study to evaluate cannabis use patterns and knowledge about cannabis legalization following passage of Prop 64. This analysis from the Impact 64 study aims to provide an overview of cannabis and driving-related knowledge, attitudes, and behaviors among California adults. For this study, we emphasized that “cannabis” use involved the use of THC-containing products, to differentiate from CBD-only cannabis products.

## Methods

The Impact 64 study consisted of three phases: (1) subject matter expert (SME) interviews with 23 individuals, including legal professionals (*n* = 3), medical providers (*n* = 8), advocates (*n* = 2), researchers (*n* = 3), individuals who use cannabis (*n* = 1), and dispensary representatives (*n* = 3); (2) an initial exploratory questionnaire of 200 people who currently use cannabis; and (3) a large-scale detailed questionnaire targeting 5000 California residents. This analysis focuses on a subset of results from the third phase regarding driving knowledge and behaviors. All study procedures were approved by the University of California San Diego Institutional Review Board (IRB).

### Questionnaire development

Partnering with Quester, a market research firm, a 25-minute study questionnaire was developed using a mixed methods study design including qualitative and quantitative questions, based on findings from the SME and exploratory questionnaire. Questionnaire content used for this sub-analysis is included in the supplement; question format included open-ended, multiple choice, and true/false style questions. The questionnaire was designed to aid in the assessment of participant engagement, such as open-ended questions and varying expected true/false responses.

### Participants and recruitment

Working with Quester’s sampling partners, the research team used quota sampling based upon the 2020 California Census to obtain a representative sample of Californians by four key demographics: gender/sex, age, race/ethnicity, and annual household income. Targeted individuals were invited to complete an initial “screener” questionnaire which collected demographics and cannabis use history; the purpose of the screener questionnaire was to identify potential participants for the full questionnaire. Participants were not aware of the study’s purpose at the time of the screener questionnaire. Inclusion criteria included residence in California, 21 years of age or above, and the ability to read English or Spanish. The sampling partners provided study participants with points that could later be redeemed for cash, gift cards, and other prizes. The points provided had a total value no greater than $10.

A subset of participants was directed to complete the full questionnaire; the full questionnaire had a goal of recruiting 5,000 participants, including 4,000 people who currently use cannabis (“current user”; self-identified as currently using cannabis and used within the past 3 months), 500 people who formerly used cannabis (“former user”; self-identified as having formerly used cannabis and has not used in 4 or more months), and 500 people who have never used cannabis (“non-user”). Selection for the full questionnaire used quota sampling and was based on demographic targets within each cannabis use group. Those employed in the cannabis, marketing/market research, or advertising/public relations industries were excluded from participation.

The official questionnaire was launched on December 2, 2022, and remained open until all target participants were recruited on February 6, 2023.

### Measures

The questionnaire materials utilized the term “cannabis” and emphasized that questions about “cannabis” use involved the use of THC-containing products, to differentiate from CBD-only cannabis products. Outcome variables included user type (those who currently use, formerly use, never used cannabis), and knowledge of Prop 64 driving-related regulations (see Table 2). Participants who currently use cannabis were also assessed for the time until participants feels safe to drive (higher risk defined as driving 3 h or less after inhalation and 5 h or less of consumption of cannabis edibles), driving-related attitudes (see Table 4), and driving behavior (“Have you ever been pulled over for driving while under the influence of cannabis”, and “Have you ever been a driver in a crash while under the influence of cannabis”).

Demographic measures included age (in years), gender (male or female; all other responses were coded as male in order to allow comparison to the binary Census measure of sex), race/ethnicity (non-Hispanic white, non-Hispanic Black or African American, Hispanic all races, and Asian/Pacific Islander; other excluded from analysis based on race/ethnicity due to very small sample size), education (high school equivalent or less, some college or college degree, and graduate degree), annual household income (less than $50,000, $50–100,000, and greater than $100,000), employment status (employed full-time, employed part-time, or not employed), marital status (single, married or has partner), presence of minors in household (present, not present), region (Northern, Central, Southern) and age of first cannabis use (in years).

Use frequency was assessed by asking “approximately how often do you use or consume cannabis, in any form, that contains THC?” Response options included less than every 6 months, once every 4–6 months, once every 2 to 3 months, 1–2 times a month, 1–3 times a week, 4–6 times a week, once a day, and multiple times a day.

### Statistical analysis

Using rake weighting (iterative proportional fitting), respondents of the screener questionnaire were weighted based on the California census for the four key demographic criteria (age group, gender/sex, race/ethnicity, annual household income). Demographic profiles of each cannabis subgroup (current, former, non-users) were identified based on this weighted screener group, establishing the target demographics for each subgroup.

Within each use subgroup of the full questionnaire, participants were weighted to match the subgroups demographics to the target demographic profile for that subgroup. As a result, the screener participant group’s demographics matched the broader California population (as defined by the 2020 Census), while each full questionnaire subgroup’s demographics match the demographics of that use group within California.

Descriptive statistics were used to explore sample characteristics, and inferential statistics (chi-square test) were performed to assess differences across knowledge and behavior groups. Multivariable analysis was utilized to evaluate relationships between key groups while adjusting for demographics (age, gender, race, education, income, employment, marital status, minors in household, region, and age of first use) with age as a continuous variable, and minors in household as dichotomous (yes/no). Statistical analysis was conducted using SPSS v. 28.0.0.0 [19] and JMP Pro v. 17.0.0 [20]. Statistical significance was assessed as *p* < 0.05.

## Results

A total of 15,208 participants completed both demographics and cannabis use history items on the screener questionnaire and were assessed for eligibility for the full questionnaire. Although region was not included as a key demographic for quota sampling or weighting, the regional distribution of the screener population approximated the 2020 California census. Weighted demographics for age group, gender/sex, race/ethnicity, and annual household income exactly matched census targets. Unweighted and target/weighted demographics of the screener questionnaire are presented in Appendix 1.

The full questionnaire was completed by 5,178 participants, including 4,020 current users, 523 former users, and 635 non-users. The remaining participants were excluded for incomplete participation, or because cannabis use or demographic quotas were already met. The participants in each use group were weighted to match their target demographics set by the screener questionnaire. Unweighted and target demographics of the full questionnaire are presented in Appendix 2. All remaining tables and information below present weighted data.

### Demographics

Of the initial questionnaire respondents who did not know the purpose of the study, 37% reported current cannabis use (use in the past 3 months), 30% formerly used cannabis, and 33% were non-users.

Demographics of each use group are summarized in Table 1. Among current users, the mean age is 41.8 years, 59% male, 38% White non-Hispanic, 82% have at least some education beyond high school, and 57% have minors in the household. 67% started using cannabis before age 25, including 33% who started at age 17 or younger (not shown in table), with a mean age of initiation of 24.4 years. 38% reported cannabis use multiple times per day, 33% use between 4 times per week to daily, and 29% are occasional users (3 times per week or less). Bivariate comparisons are shown in Table 1.

Adjusting for other demographics in multivariate analysis, current users were more likely than former or non-users to be younger, male, Hispanic (any race) or Black non-Hispanic (*p* < 0.001), and working full time (not shown in table). Additional demographic analysis is published elsewhere [21].

**Table 1:** Demographics of the participants of the impact 64 full questionnaire by cannabis use history

	Current users (A) *n* = 4020 Weighted %	Former users (B) *n* = 523 Weighted %	Non-users (C) *n* = 635 Weighted %
Age			
Mean (SD)	41.8 (14.0) ^BC^	48.2 (16.1) ^C^	52.5 (15.8)
Gender			
Male	58% ^BC^	47% ^C^	40%
Female (ref)	41% ^BC^	53% ^C^	60%
Genderqueer, agender, transgender, or other	1%	< 0.5%	< 0.5%
Race/Ethnicity			
White non-Hispanic	38% ^B^	44% ^C^	39%
Hispanic (all races)	42% ^BC^	35% ^C^	30%
Black non-Hispanic	8% ^BC^	5%	5%
Asian/Pacific Islander (ref)	11% ^BC^	14% ^C^	25%
Educational status			
High school diploma or lower	18%	15%	17%
Some college or college degree	68% ^C^	69% ^C^	64%
Graduate degree (ref)	14%	15%	19%
Annual Household income			
Under 50 K	24% ^C^	26%	28%
50–100 K (ref)	28%	29%	30%
Greater than 100k	48% ^C^	45%	42%
Employment status			
Employed full-time (ref)	64% ^BC^	50%	45%
Employed part-time	13%	14%	14%
Not employed	23% ^BC^	35%	41%
Marital status			
Single (ref)	45% ^B^	49% ^C^	43%
Married or has partner	55% ^B^	51% ^C^	57%
Minors in household			
No minors in household (ref)	43% ^BC^	49%	53%
Minors in household	57%	51%	47%
Age 0–6	19%	19%	15%
Age 7–12	27%	18%	15%
Age 13–17	21%	15%	15%
Residence/Region			
Northern region	26%	27%	24%
Central region	16%	17%	15%
Southern region (ref)	58%	57%	61%
Age of first cannabis use			
Mean (SD)	24.4 (13)	23.6 (13)	--
Use frequency			
Occasional use (3 times per week or less)	29%	--	--
Frequent use (4 times per week to daily)	33%	--	--
Very frequent use (more than once daily)	38%	--	--
Prop 64 familiarity			
Very familiar	32%^BC^	12%	13%
Somewhat familiar	40%	44%	41%
Have heard of it, but don’t know much about it	22% ^BC^	33%	37%
Have never heard of it	6% ^BC^	10%	9%

Significance was determined using bivariate analysis by comparing each cannabis use category

Annotations (^A, B, C^) show statistical significance using p-value < 0.05, where the use groups (columns A, B, C) differed from each other use group

### Knowledge about prop 64 driving regulations

Table 2 summarizes knowledge of Prop 64 regarding driving and driving under the influence. 63% of participants in all groups were aware that drivers and passengers cannot smoke or ingest cannabis products in a moving vehicle. Few participants (12–15%) incorrectly reported that it is legal to smoke cannabis in the car as a passenger. Most participants correctly identified that you could get a DUI for driving under the influence of cannabis; former users were significantly more likely (77%), compared to current (73%, *p* < 0.001) or non-users (67%, *p* < 0.001) to report this as true. A small majority (51–59%) of participants knew that any container of cannabis inside a moving vehicle must be unopened and sealed.

The knowledge of current users was further analysed using multivariable analysis. After correcting for other demographic variables, current users who knew that drivers and passengers cannot use cannabis in a moving vehicle were more likely female, white, and have at least some college. Current users who knew one could get a citation for DUIC were more likely to be white, have at least some college, and have no minors in the household. Current users who knew that cannabis must be unopened and sealed in a vehicle were more likely to be female and white.

**Table 2:** Knowledge about prop 64 driving regulations

	Current users *n* = 4020 %	Former users *n* = 523 %	Non-users *n* = 635 %	Demographical analysis ~ Current users’ odd ratio with confidence interval (comparing stated use frequency vs. other use frequencies)
*Which of the following are you aware that are true under the Prop 64 law?*				
Drivers and passengers cannot smoke or ingest cannabis products in a moving vehicle	62%	64%	63%	Female (AOR 1.2, CI 1.069–1.398), White (vs. Hispanic all races, AOR 1.6, CI 1.421–1.951), and have at least some college degree (vs. High school or less, AOR 1.3, CI 1.044–1.475).
*Which of the following are TRUE regarding Prop 64?* ^*a*^	% correct	% correct	% correct	
It is legal to smoke cannabis in the car as a passenger (Not true)	86%	88%	85%	NS
You can get a DUI for driving under the influence of cannabis (True)	73%^BC^	78%^C^	67%	Female (AOR 1.2, CI 1.035–1.391), White (vs. Hispanic all races, AOR 1.7, CI 1.389–1.976), have at least some college degree (vs. High school or less, AOR 1.3, CI 1.001–1.475), and have no minors in the household (vs. minors, AOR 1.3, CI 1.150–1.553)
Any container of cannabis inside a moving vehicle must be unopened and sealed, just like alcohol (True)	59%^C^	55%	51%	Female (AOR 1.3, CI 1.158-1.500) and White (vs. Hispanic all races, AOR 1.2, CI 1.053–1.428)

~Odd ratio and confidence interval using multivariable analysis. E.g., Compared to those who did not believe “Drivers and passengers cannot smoke or ingest cannabis products in a moving vehicle” those that did were more likely to be female, white, and at least have college degree.

*NS* Not Significant

ABC Significant difference between that column and the associated column (A, B, or C) by Chi-squared test, with *p* < 0.001

^a^ Correct responses are notes in parentheses

### Time until feels safe to drive after use of cannabis

Table 3 shows the reported time until it feels safe to drive after flower inhalation and edibles for select demographics with chi squared comparisons. The mean time to feel safe to drive was 2.4 h for flower-type products and 3.7 h for edibles. With respect to flower inhalation, 34% felt safe to drive within an hour or less of use, with an additional 30% feeling safe within 2–3 h, totalling 64% in this higher risk category. For edibles, 19% felt save to drive within 1 h of use, an additional 19% between 2 and 3 h, and 17% between 4 and 5 h of use, totalling 55% of all participants in the higher risk category.

**Table 3:** Time until it feels safe to drive for inhalation of cannabis, by select demographics

	Time until it feels safe to drive after INHALATION of flower cannabis							
	Higher Risk			Lower Risk				*p*-value^+^
	1 h or less *n* = 618	2–3 h *n* = 542	Total Higher Risk	4–5 h *n* = 240	6 + hr, same day *n* = 125	Next day *n* = 293	Total Lower Risk	
All (*n* = 1818)^a^	34%	30%	64%	13%	7%	16%	36%	--
Age								
21–25	33%	29%	62%	14%	10%	13%	38%	0.538
26–35	36%	31%	67%	11%	8%	14%	33%	0.040
36–45	39%	29%	68%	14%	6%	12%	32%	0.042
46–55	30%	34%	64%	14%	6%	15%	36%	0.783
56–65	28%	25%	54%	17%	4%	25%	46%	0.002
66 +	26%	22%	48%	14%	6%	31%	52%	0.001
Gender								
Male	36%	29%	65%	14%	7%	14%	35%	--
Female	30%	31%	61%	12%	6%	20%	39%	0.095
Use type								
Recreational only	34%	30%	64%	12%	6%	18%	36%	0.982
Both	36%	30%	66%	13%	8%	13%	34%	0.085
Medicinal only	24%	31%	55%	17%	6%	22%	45%	0.007
Use frequency								
Occasional	16%	27%	43%	20%	10%	27%	57%	< 0.001
Frequent	30%	32%	63%	10%	8%	19%	37%	0.520
Very frequent	44%	29%	74%	12%	5%	9%	26%	< 0.001

	Time until it feels safe to drive after use of cannabis EDIBLE							
	Higher Risk				Lower Risk			p-value^+^
	1 hour or less *n* = 618	2–3 hour *n* = 542	4–5 hour *n* = 240	Total Higher Risk	6 + hours, same day *n* = 125	Next day *n* = 293	Total Lower Risk	
All (*n* = 1887)^a^	19%	19%	17%	55%	14%	31%	45%	--
Age								
21–25	20%	19%	19%	58%	13%	29%	42%	0.33
26–35	20%	21%	19%	61%	12%	27%	39%	< 0.001
36–45	23%	20%	18%	60%	17%	23%	40%	0.01
46–55	16%	20%	18%	55%	12%	33%	45%	0.9
56–65	14%	14%	15%	44%	17%	39%	56%	< 0.001
66 +	12%	8%	7%	26%	12%	61%	74%	< 0.001
Gender								
Male	21%	20%	19%	61%	13%	56%	39%	--
Female	15%	17%	15%	47%	15%	38%	53%	< 0.001
Use type								
Recreational only	21%	21%	17%	60%	11%	29%	40%	0.003
Medicinal only	14%	13%	14%	42%	14%	45%	58%	< 0.001
Both	19%	19%	18%	56%	17%	27%	44%	0.5
Use frequency								
Occasional use	9%	17%	15%	41%	14%	45%	59%	< 0.001
Frequent use	17%	21%	18%	56%	14%	31%	44%	0.7
Very frequent use	30%	19%	19%	67%	14%	19%	33%	< 0.001

^a^Shows % of current users who provided a response; excludes non-drivers (inhalation = 162, 7%; edibles n = 147, 6%) and responders of “it depends” (inhalation n = 286, 13%; edibles n = 242, 11%)

^+^Statistical significance of by Chi-squared comparison of the “total lower risk” versus the “total higher risk”

In multivariable analysis including all demographics (not shown in table), the odds of being in the higher-risk wait time category for flower inhalation (3 or fewer hours after use) compared to those in the lower risk wait time category (more than 3 h) was greater in those who started cannabis at 17 or younger (vs. 45+, AOR 6.4, CI 3.964–10.320). There were no other significant differences by demographics. Those in the higher-risk wait time category after cannabis edible use (5 or fewer hours after consumption) were more likely than those is the lower-risk category (6 or more hours) to be male (vs. female, AOR 1.6, CI 1.347–1.979), income under 50k (vs. 50-100k, AOR 1.5, CI 1.109–2.008), married or partnered (vs. not married or partnered, OR 1.4, CI 1.122–1.711), had minors in household (vs. no minors in household, AOR 1.4, CI 1.143–1.691), and started cannabis 17 or younger (vs. 45+, AOR 2.8, CI 1.908–4.171).

### Driving behaviors and attitudes

Participants who currently use cannabis also reported specific driving-related behaviors and attitudes which are summarized in Table 4. Most respondents reported that legalization either had no effect (65%) or decreased (21%) the Likelihood that they would drive under the influence of cannabis, while 14% of current users report an increased Likelihood. 23% correctly knew that “to the best of my knowledge, I can drive after using cannabis as long as I’m not impaired.”

Regarding being a passenger in the car with an impaired driver, 38% of current users reported riding in a car with a driver who had recently used cannabis. Of those, 89% felt safe doing so. Finally, current users reported utilizing a variety of other modes of transportation after using cannabis, including safer legal options such as public transportation (26%), taxi (42%), and designated driver (53%). However, 8% reported use of a motorized scooter or bike while using cannabis.

Multivariable analysis was done to further evaluate higher risk attitudes by demographics. After correcting for other demographics, current users who reported an increased likelihood of driving under the influence of cannabis since passage of Prop 64 were more likely to be male, Hispanic, employed, and have minors in the household. Participants who reported feeling safe while riding in a car with a driver who had recently used cannabis were more likely to be male, Black non-Hispanic, employed, and started cannabis before age 17.

**Table 4:** Driving behaviors and attitudes among participants who currently use cannabis

	Current users *n* = 4020	Demographical analysis~ Odd ratio with confidence interval (comparing stated use frequency vs. other use frequencies)
*Has the legalization of cannabis affected your likelihood of driving under the influence*		
It increased the likelihood	14%	Male (AOR 1.6, CI 1.294–1.953), Hispanic (vs. white non-Hispanic, AOR 1.7, CI 1.345–2.125), employed (AOR 1.8, 1.453–2.804), and have minors in the household (AOR 1.7, CI 1.375–2.095)
It decreased the likelihood	21%	NS
No change	65%	NS
*In the past 3 months*,* did you ride in a car with a driver who has recently used cannabis*		
Yes, and I felt safe	34%	Male (AOR 1.3, CI 1.149–1.534), Black non-Hispanic (vs. Asian, AOR 1.5, CI 1.098–2.098), employed (vs. unemployed, AOR 1.4, CI 1.159–1.689), and started cannabis before age 17 (vs. who started at age 45 or older, AOR 4.3, CI 3.046–6.178).
Yes, and I felt unsafe	4%	NS
No	54%	NS
Not sure	8%	NS
*To the best of my knowledge*,* I can drive after using cannabis as long as I’m not impaired*		
True	23%	NS
False	64%	NS
Don’t know	13%	NS
*What other modes of transportation do you take when using cannabis?*		
Public transportation	26%	NS
Ride sharing or taxi	42%	NS
Friends/designated driver	53%	NS
Motorized scooter or bike	8%	NS
Other	2%	NS
None	26%	NS

~Odd ratio and confidence interval using multivariable analysis. E.g., Compared to other groups combined, those who reported that legalization increased their likelihood of DUIC were more likely to be male, Hispanic, employed, and have minors in the household

*NS *Not Significant

### Driving outcomes and attitudes

Among participants who currently use cannabis, 9% reported ever being pulled over for driving while under the influence of cannabis, and 7% reported having been a driver in a crash while under the influence of cannabis. These are higher rates than reported among those who formerly used cannabis (3% and 4%, respectively). Of those who currently use cannabis who had been involved in a crash, 70% had been pulled over for driving while under the influence of cannabis at some point. Among those who currently use cannabis who were pulled over, 52% had also used alcohol or other drugs in addition to cannabis at the time of being pulled over. Among those who currently use cannabis who were the driver in a crash, 59% had used alcohol or other drugs in addition to cannabis at the time of the crash.

Attitudes about cannabis use and driving outcomes among current users are summarized in Table 5. Those who stated it was legal to smoke cannabis as a passenger in a car were 2–3 times more Likely to be stopped or get in a crash while under the influence of cannabis. Those who stated they could drive after cannabis use if not impaired were four times more Likely to be stopped and almost 6 times more likely to get in a crash. Conversely, those who correctly identified that drivers and passengers cannot use cannabis in a moving vehicle and those who correctly knew you could get a DUI arrest for driving under the influence, were significantly less likely to be stopped or be in a crash. Finally, having a higher-risk wait time (≤ 3 h for inhalation or ≤ 5 h for edibles), was associated with having been stopped and having been in a crash while under the influence of cannabis.

**Table 5:** Outcomes and driving under the influence among people who currently use cannabis

		Have ever been pulled over for driving while under the influence of cannabis *n* = 368 (9%)	Have ever been a driver in a crash while under the influence of cannabis *n* = 269 (7%)
It is legal to smoke cannabis in a car as a passenger	Yes (*n* = 564)	15.4%**^a^	13.3%**
	No (*n* = 3456)	8.1%	5.6%
I can drive after using cannabis as long as I’m not impaired	Yes (*n* = 919)	19.9%**	16.0%**
	No (*n* = 2584)	4.2%	2.7%
Drivers and passengers cannot smoke or ingest cannabis products in a moving vehicle	True (*n* = 2502)	7.1%**	4.7%**
	False (*n* = 1518)	12.6%	9.9%
One can get DUI if driving under influence of cannabis	Yes (*n* = 2917)	6.7%**	4.5%**
	No *n* = 1103	15.6%	12.4%
Time until feels safe to drive after flower inhalation	3 h or less (*n* = 1160)	17.5%**	12.6%**
	4 or more hours (*n* = 658)	6.1%	5.7%
Time until feels safe to drive after edibles	5 h or less (*n* = 1038)	16.3%**	13.0%**
	6 h or more (*n* = 849)	3.2%	2.0%
In the past 3 months, did you ride in a car with a driver who has recently used cannabis?	Yes (*n* = 1527)	20.6%**	15.1%**
	No (*n* = 2170)	1.7%	1.4%

Row responses are compared using Chi squared analysis, and significance is indicated is indicated by * for P<0.05 and ** for p<0.001. For example, of the 564 participants who believed it was legal to smoke cannabis in a car as a passenger, 15% had been pulled over and 13% had been involved in a crash. Those who believed it is legal to smoke cannabis in a car as a passenger were significantly more likely than those who did not believe it was legal to smoke cannabis in a car as a passenger to report having been pulled over (15% vs 8%, p<0.001) or been involved in a crash (13% vs 6%, p<0.001)

^a^ Percentages indicate the % of participants from each row who said they had reported being pulled over (column A) or in a crash (column B)

## Discussion

Six years after the legalization of adult/recreational cannabis use via Prop 64, California adults demonstrate mixed awareness and knowledge of the driving-related legalities of cannabis use. Among participants who currently use cannabis, 38% did not know it is unlawful for drivers and passengers to smoke or ingest cannabis in a moving vehicle, and 26% were not aware that driving under the influence of cannabis can result in a DUI citation/arrest. This relative lack of awareness poses significant legal and social risks to individuals in addition to public health and safety concerns. Lower knowledge of driving-related laws and reported higher risk behavior such as lower reported time before feeling safe to drive was associated with both a history of being pulled over and involvement as a driver in a crash while under the influence of cannabis. Targeted interventions should focus on the cannabis using populations with lower knowledge of these laws.

Regardless of regulations, some drivers tend to overestimate their ability to drive safely after cannabis use. Simulator-based and on-road studies have shown that cannabis use can impair some drivers for 3–4 h after smoking [22, 23] and longer for ingestion [24]. In some cases, participants felt ready to drive 1.5 h after ingestion, despite ongoing performance impairments [22]. Other studies suggest waiting at least six hours after cannabis use before driving [25]. However, in this study, current users reported an average time of only 2.4 h for inhalation and 3.7 h for ingestion before feeling safe to drive. Unlike alcohol, THC concentrations do not directly correlate to impairment, and there is significant inter-individual variability in response to THC [26]. In addition, dosage, product type, and route of administration all impact an individual’s onset and duration of intoxication, making it difficult to communicate universal wait time recommendations to the public. The absence of available biological measures of intoxication further complicates assessment. Future research should explore the effect of various product types, particularly high-THC concentrates (e.g., dabbing, vaping) on driving impairment.

Some studies in other states and countries have shown increases in crash and DUI risk following cannabis legalization, but others have found reduced self-reported DUIC in cannabis-legalized states [12, 27]. Few of the people who currently use cannabis (12.8%) in the current study reported that the passage of Prop 64 increased their likelihood of DUIC. Self-reported increased risk for DUIC could be secondary to perceived permissiveness of DUIC or related to increased access and opportunity. Individuals who were unaware of DUIC law were more likely to be pulled over, highlighting the importance of public education. Second, participants who reported they can drive after using cannabis if not impaired were more likely to have been pulled over or involved in a crash while under the influence of cannabis. This suggests a possible gap between actual and perceived impairment and indicates poor understanding of state laws, contributing to higher risk behaviors.

In addition to the direct risk associated with driving, participants are putting themselves at risk in other ways. 8% of those who currently use cannabis reported riding a motorized scooter or bike while under the influence, behavior which presents legal and safety risks. Passenger behavior is also noteworthy; almost 40% of current cannabis users reported riding within the last 3 months with a driver who had recently used cannabis. This is higher than the 10–20% rate shown in prior studies of adults [28] and youth [29, 30]. Most of the participants felt safe as a passenger, suggesting an underestimation of the risks of riding with someone who may be under the influence. Prior studies have also demonstrated an association between DUIC as a driver, and riding as a passenger with an impaired driver [31, 32]. Consistent with this, participants who reported riding as a passenger with a driver who had recently used cannabis were far more likely to report prior citation or crash as a driver while under the influence of cannabis.

Engagement in high-risk driving behaviors is a complex and nuanced problem which will not easily be mitigated. This study shows mixed awareness and knowledge of driving-related cannabis restrictions since the passage of Prop 64 to legalize recreational cannabis in California. Participants with decreased knowledge on multiple driving-related restrictions of Prop 64 were more likely to engage in related risky behaviors. Future research should continue assessing the public’s knowledge about cannabis legalization laws to help identify potential high-risk groups and specific knowledge gaps in each region nationwide. Interventions aimed at reducing driving-related adverse events should consider educational campaigns regarding existing regulations.

A strength of this study includes the mixed method study design as well as the large sample size matched to the California 2020 census demographics, which allows for reasonable generalization to the cannabis-using population in California. The emphasis on THC-containing cannabis products (versus CBD-only products) allows for a focused assessment of THC-related behaviors, although THC dosing was not assessed. Participation was anonymous which increases disclosure of high-risk use behaviors and limits reporting bias of self-reported behavior which may not be legal. Limitations include the exclusion of individuals under age 21 (the legal limit in California for recreational use), which limits insights into younger individuals, and use of an online questionnaire which excludes participants without access to technology. As a cross-sectional study, causation, and changes in findings over time cannot be assessed. Some driving outcomes were assessed as lifetime events, which may not be generalizable to post-legalization behavior. Finally, there is reporting bias associated with the questionnaire-based evaluation of behavior.

## Conclusion

Knowledge of driving-related cannabis restrictions in California is variable. Lower awareness, reduced risk perception, and shorter driving wait times after cannabis use were associated with negative driving outcomes (e.g., being pulled over, involved in a crash while under the influence of cannabis). Educational campaigns should focus on improving public understanding of driving laws, promotion of safe driving wait times, and raising awareness of the risks of driving under the influence of cannabis.

## Supplementary Information

Supplementary Material 1.

Supplementary Material 2. Appendix 1: Demographics of population by California census/target vs. demographics of unweighted/actual study population for screener questionnaire. Appendix 2: Demographics of census-weighted/target current users vs. demographics of unweighted/actual current users

## Data Availability

The datasets generated and/or analysed during the current study are not publicly available due to ongoing data analysis but may be available from the corresponding author on reasonable request.

## Abbreviations

AOR: Adjusted Odds Ratio
DUI: Driving Under the Influence
DUIC: Driving Under the Influence of Cannabis
Prop 64: Proposition 64
DCC: Department of Cannabis Control
IRB: Institutional Review Board
SME: Subject Matter Expert
THC: Delta 9 Tetrahydrocannabinol
