# Supplementary material for: Driving and cannabis use: a questionnaire about knowledge and behaviors after the legalization of recreational cannabis in California
Source: BMC Public Health. 2025 Sep 30;25:3219. doi: 10.1186/s12889-025-24309-4 (PMC12487619; doi:10.1186/s12889-025-24309-4)
Supplement: Supplementary file 1 — Supplementary Material 1. [file 12889_2025_24309_MOESM1_ESM.docx]

Cannabis Use and Driving: A Questionnaire About Knowledge, Attitudes and Behaviors After the Legalization of Recreational Cannabis in California

Selected questions from study questionnaire

April 2025

-----INITIAL/SCREENER QUESTIONNAIRE-----

S0Industry

**Type:** Checkbox

Do you or does anyone in your household work in any of the following types of businesses or occupations? Please select all that apply.

Randomize

| Opt. # | Option Text | Notes |
| --- | --- | --- |
| 1 | Advertising/Public Relations | Terminate |
| 2 | Financial Services |  |
| 3 | Food/Beverage |  |
| 4 | Marketing/Marketing Research | Terminate |
| 5 | Real Estate/Construction |  |
| 6 | Sales/Sales Promotion |  |
| 7 | Cannabis Industry | Terminate |
| 8 | None of the above | Anchor & Mutually Exclusive |

S1aZipCode

**Type:** Digits

What is your residential zip code?

S1bState

**Type:** Single Select Dropdown

In which state do you live?

Must be California, else Terminate

S2aAge

**Type:** Digits

What is your age?

_____ Allow 1-99; Must be 21+, else Terminate

S3Gender

**Type:** Radio Button

What is your gender?

| Opt. # | Option Text | Notes |
| --- | --- | --- |
| 1 | Male |  |
| 2 | Female |  |
| 3 | Genderqueer or non-binary |  |
| 4 | Agender |  |
| 5 | Not specified above |  |
| 6 | Prefer not to answer |  |

S4aHispanic

**Type:** Radio Button

Are you of Spanish, Hispanic, or Latino background or origin? This includes Mexican, Mexican American, Puerto Rican, Cuban, and all other Spanish, Hispanic, or Latino origins.

| Opt. # | Option Text | Notes |
| --- | --- | --- |
| 1 | Yes |  |
| 2 | No |  |
| 3 | Prefer not to answer |  |

S4bRace

**Type:** Checkbox

Are you…? Select all that apply.

| Opt. # | Option Text | Notes |
| --- | --- | --- |
| 1 | White/Caucasian |  |
| 2 | Black/African American |  |
| 3 | American Indian/Native American |  |
| 4 | Asian |  |
| 5 | Pacific Islander |  |
| 6 | Some other race |  |
| 7 | Prefer not to answer | Exclusive |

S5bHHIncome

**Type:** Radio Button

And what is your total annual household income (including others in your household) before taxes?

| Opt. # | Option Text | Notes |
| --- | --- | --- |
| 1 | Less than $25,000 | Hide if S5a = 2-8 |
| 2 | $25,000 to $49,999 | Hide if S5a = 3-8 |
| 3 | $50,000 to $74,999 | Hide if S5a = 4-8 |
| 4 | $75,000 to $99,999 | Hide if S5a = 5-8 |
| 5 | $100,000 to $124,999 | Hide if S5a = 6-8 |
| 6 | $125,000 to $149,999 | Hide if S5a = 7-8 |
| 7 | $150,000 to $249,999 | Always show |
| 8 | $250,000 or more | Always show  Autopunch if S5a = 8 |

D2HHSize

**Type:** Radio Button

How many people are living or staying at your current address? (Include yourself and any other adults or children who are living or staying at this address for at least two months)

| Opt. # | Option Text | Notes |
| --- | --- | --- |
| 1 | 1 |  |
| 2 | 2 |  |
| 3 | 3 |  |
| 4 | 4 |  |
| 5 | 5 |  |
| 6 | 6 |  |
| 7 | 7 |  |
| 8 | 8 or more |  |
| 9 | Prefer not to answer |  |

D2aHHComposition

**Type:** Checkbox

And just to be sure I understand, thinking of who are living or staying at your current address, are any of them … ?

| **Opt. #** | **Option Text** | **Notes** |
| --- | --- | --- |
| 1 | Under age 6 |  |
| 2 | 7 to 12 years old |  |
| 3 | 13 to 17 years old |  |
| 4 | My adult children age 18+ |  |
| 5 | My parents or in-laws |  |
| 6 | Spouse or partner |  |
| 7 | My grandchildren |  |
| 8 | Grandparents |  |
| 98 | Other family member (aunts, uncles, cousins) |  |
| 9 | Roommate(s) |  |
| 99 | Prefer not to answer |  |

S7Usage

**Type:** Radio

Which of the following statements best describes you?

Please note: THC (tetrahydrocannabinol) is the substance in cannabis products that is responsible for the ‘high’ or ‘stoned’ feeling when using cannabis. Pure CBD products do not contain THC.

| Opt. # | Option Text | Notes |
| --- | --- | --- |
| 1 | I currently use or consume **cannabis that contains THC** (in any form) |  |
| 2 | I do not currently use or consume **cannabis that contains THC** (in any form), but I have in the past |  |
| 3 | I have never used or consumed **cannabis that contains THC** (in any form) | Qualifies for Non-User |

Ask if S7Usage=1 or 2 (Currently use, or used in the past)

S8LastConsumed

**Type:** Radio

When was the last time you used or consumed **cannabis that contains THC**, in any form?

| Opt. # | Option Text | Notes |
| --- | --- | --- |
| 1 | Today | May quality for Current User |
| 2 | Within the past week | May quality for Current User |
| 3 | Within the past month | May quality for Current User |
| 4 | Within the past 2-3 months | May quality for Current User |
| 5 | Within the past 4-6 months | May quality for Former User |
| 6 | Within the past 7-12 months | May quality for Former User |
| 7 | More than 12 months ago | May quality for Former User |

Ask if S7Usage=1 or 2 (Currently use, or used in the past)

S9UseFrequency

**Type:** Radio

Ask Current Users (S7=1): Approximately how often do you use or consume cannabis, in any form, that contains THC?

Ask Past Users (S7=2): Approximately how often did you use or consume cannabis, in any form, that contains THC **in the past**?

| Opt. # | Option Text | Notes |
| --- | --- | --- |
| 1 | Multiple times a day | May qualify for current or former user |
| 2 | Once a day | May qualify for current or former user |
| 3 | 4-6 times a week | May qualify for current or former user |
| 4 | 1-3 times a week | May qualify for current or former user |
| 5 | 1-2 times a month | May qualify for current or former user |
| 6 | Once every 2 to 3 months | May qualify for current or former user |
| 7 | Once every 4 to 6 months | Terminate |
| 8 | Less than every 6 months | Terminate |

----SELECT QUESTIONS FROM FULL QUESTIONNAIRE----

Ask if Current User or Former User

Q1AgeUse

**Type:** Digits

At what age did you start regularly/consistently using cannabis?

Ask if Current User or Former User

Q2aMedRec

**Type:** Checkbox

IF QA1Assigned=1 show:

Please select **all the reasons** you currently use cannabis from the list below.

IF QA1Assigned=2 show:

Please select **all the reasons** you used to use cannabis from the list below.

Randomize

| Opt. # | Option Text | Notes |
| --- | --- | --- |
| 1 | Medicinal purposes |  |
| 2 | Recreational purposes |  |
| 3 | Religious or spiritual purposes |  |
| 98 | Other (please specify) | Anchor |

**Ask if Current User**

**Q15bProductsUseCurrent**

**Type:** Checkbox

Currently, which of the following cannabis products do you use regularly? Please select all that apply.

| **Opt. #** | **Option Text** | **Notes** |
| --- | --- | --- |
| 1 | Dried flower (i.e., smoking, vaporized flower) |  |
| 2 | Oils/tinctures |  |
| 3 | Edibles or capsules |  |
| 4 | Vaping concentrates | **Keep together** |
| 5 | Dabbing concentrates |  |
| 6 | Topical/transdermal (i.e., creams, lotions, salves, patches) |  |
| 7 | Beverages |  |
| 8 | Other forms of THC (Delta 8, Delta 10, THC-V) | **Anchor** |
| 98 | Other (specify) | **Anchor, Always Show** |

Ask everyone

Q33Familiarity

**Type:** Radio

How familiar are you with Prop 64, the law that legalized recreational use of cannabis in California in 2016?

| Opt. # | Option Text | Notes |
| --- | --- | --- |
| 1 | Very familiar |  |
| 2 | Somewhat familiar |  |
| 3 | Have heard of it, but don’t know much about it |  |
| 4 | Have never heard of it |  |

Ask everyone

Q41Knowledge

**Type:** Check Box

Which of the following are TRUE regarding Prop 64? Select all that apply.

Randomize

| Opt. # | Option Text | Notes |
| --- | --- | --- |
| 1 | It’s legal to smoke cannabis in the car as a passenger |  |
| 2 | You can get a DUI for driving under the influence of cannabis |  |
| 3 | Individual cities can set their own regulations regarding driving after using cannabis |  |
| 4 | Any container of cannabis inside a moving vehicle must be unopened and sealed, just like alcohol |  |
| 99 | None of the above | Anchor, Exclusive |

Ask everyone

Q50Awareness1

**Type:** Check Box

Which of the following are you aware are legal for adults 21+ under the Prop 64 law? Select all that apply.

Randomize

| Opt. # | Option Text | Notes |
| --- | --- | --- |
| 1 | Possess 1 ounce or less of recreational cannabis and a quarter of an ounce or less of cannabis concentrate |  |
| 2 | Use cannabis on private property, but not in public places, such as bars or restaurants |  |
| 3 | Give away 1 ounce or less of recreational cannabis and a quarter of an ounce or less of cannabis concentrate to other adults 21 years and older without compensation |  |
| 4 | Transport 1 ounce or less of recreational cannabis and a quarter of an ounce or less of cannabis concentrate cannabis in the trunk of the vehicle |  |
| 99 | None of the above | Anchor, Exclusive |

Ask everyone

Q51Awareness2

**Type:** Check Box

Which of the following are you aware that are true under the Prop 64 law? Select all that apply.

Randomize

| Opt. # | Option Text | Notes |
| --- | --- | --- |
| 1 | A person cannot smoke cannabis in places where it is illegal to smoke tobacco |  |
| 2 | Drivers and passengers cannot smoke or ingest cannabis products in a moving vehicle |  |
| 3 | Prop 64 does not decriminalize cannabis use for minors, but it does reduce the maximum penalty for most cannabis-related offenses to an infraction (except for manufacturing and driving under the influence of cannabis) |  |
| 4 | A person currently serving a sentence for a conviction of an eligible cannabis-related offense may petition the court for resentencing or dismissal of eligible convictions |  |
| 99 | None of the above | Anchor, Exclusive |

Ask everyone

Q77DrivingPerceptionsDUI

**Type:** Radio

Has the legalization of cannabis affected your likelihood of **driving under the influence of alcohol**?

| Opt. # | Option Text | Notes |
| --- | --- | --- |
| 1 | Yes, it increased the likelihood |  |
| 2 | Yes, it decreased the likelihood |  |
| 3 | No change |  |

Ask if Current User

Q69DrivingPreceptionsSafety

**Type:** Radio

To the best of your knowledge, is the following true or false regarding Prop 64?

I can drive after using cannabis as long as I'm not impaired.

| Opt. # | Option Text | Notes |
| --- | --- | --- |
| 1 | True |  |
| 2 | False |  |
| 3 | Don’t know | Anchor |

Ask if Current User + flower use

Q72aDrivingWaitInhale

**Type:** Radio

After you inhale **cannabis flower**, how long do you usually wait until you feel safe to drive?

| Opt. # | Option Text | Notes |
| --- | --- | --- |
| 1 | Immediately |  |
| 2 | 30 minutes |  |
| 3 | 1 hour |  |
| 4 | 2 hours |  |
| 5 | 3 hours |  |
| 6 | 4 hours |  |
| 7 | 5 hours |  |
| 8 | 6 hours |  |
| 9 | 7 hours |  |
| 10 | 8 or more hours |  |
| 11 | I wait until the next day |  |
| 12 | Not sure / it depends |  |
| 13 | I’m a non-driver |  |

Ask if Current User + edible use

Q72bDrivingWaitEdible

**Type:** Radio

After you use a **cannabis edible**, how long do you usually wait until you feel safe to drive?

| Opt. # | Option Text | Notes |
| --- | --- | --- |
| 1 | Immediately |  |
| 2 | 30 minutes |  |
| 3 | 1 hour |  |
| 4 | 2 hours |  |
| 5 | 3 hours |  |
| 6 | 4 hours |  |
| 7 | 5 hours |  |
| 8 | 6 hours |  |
| 9 | 7 hours |  |
| 10 | 8 or more hours |  |
| 11 | I wait until the next day |  |
| 12 | Not sure / it depends |  |
| 13 | I’m a non-driver |  |

Ask if Current User or Former User

Q74EverDUICrash

T**ype:** Radio Grid

Please indicate if you have experienced the following situations.

Scale/Columns

| Opt. # | Option Text | Notes |
| --- | --- | --- |
| 1 | Yes |  |
| 2 | No |  |

Rows - Randomize

| Opt. # | Option Text | Notes |
| --- | --- | --- |
| 1 | Have you ever been pulled over for driving while under the influence of cannabis? |  |
| 2 | Have you ever been a driver in a crash while under the influence of cannabis? |  |

Ask if Q74_1 or Q74_2 = Yes

Q75bCrash

**Type:** Checkbox

When you were pulled over or in a crash, had you used alcohol or other drugs in addition to cannabis?

| Opt. # | Option Text | Notes |
| --- | --- | --- |
| 1 | Yes |  |
| 2 | No |  |

Ask if Current User

Q75Trasportation

**Type:** Checkbox

Which, if any, other modes of transportation do you take when using cannabis? Please select all that apply.

Randomize

| Opt. # | Option Text | Notes |
| --- | --- | --- |
| 1 | Public transportation |  |
| 2 | Ride sharing (e.g., Uber, Lyft) or taxi |  |
| 3 | Friends/designated driver |  |
| 4 | Motorized scooter or bike |  |
| 98 | Other (specify) | Anchor |
| 99 | None | Anchor, Exclusive |

Ask everyone

Q78RidingWithUser

**Type:** Radio Button

In the past 3 months, did you ride in a car with a driver who has recently used cannabis?

| Opt. # | Option Text | Notes |
| --- | --- | --- |
| 1 | Yes, and I felt safe |  |
| 2 | Yes, but I did not feel safe |  |
| 3 | No |  |
| 4 | Not sure |  |

-----ADDITIONAL DEMOGRAPHICS----

D1Marital

**Type:** Radio Button

What is your marital status?

| Opt. # | Option Text | Notes |
| --- | --- | --- |
| 1 | Single, never married |  |
| 2 | Living with partner |  |
| 3 | Married |  |
| 4 | Widowed |  |
| 5 | Divorced/Separated |  |
| 6 | Prefer not to answer |  |

D3Education

**Type:** Radio Button

What is the highest degree or level of school you have completed?

| **Opt. #** | **Option Text** | **Notes** |
| --- | --- | --- |
| **Category Header** | Education through Grade 12 |  |
| 1 | Less than 9th grade |  |
| 2 | 9th to 12th grade, no diploma |  |
| **Category Header** | High School Graduate or Equivalent |  |
| 3 | Regular High School Diploma |  |
| 4 | GED or alternative credential |  |
| **Category Header** | Some College or College Degree |  |
| 5 | Occupational trade program |  |
| 6 | Some college credit, but no degree |  |
| 7 | Associate's degree (for example:  AA, AS) |  |
| 8 | Bachelor's degree (for example:  BA, BS) |  |
| **Category Header** | Graduate or Professional Degree |  |
| 9 | Master's degree (for example:  MA, MS, MEng, MEd, MBA) |  |
| 10 | Professional degree (for example:  MD, DDS, DVM, LLB, JD) |  |
| 11 | Doctorate degree (for example:  PhD, EdD) |  |

D4Employment

**Type:** Check Box

Which of the following best describes your current employment status?

| Opt. # | Option Text | Notes |
| --- | --- | --- |
| 1 | Employed – full-time |  |
| 2 | Employed – part-time |  |
| 3 | Self-Employed – full-time |  |
| 4 | Self-Employed – part-time |  |
| 5 | Retired |  |
| 6 | Student – full-time |  |
| 7 | Student – part-time |  |
| 8 | Military |  |
| 9 | Full-time parent, homemaker |  |
| 10 | Not currently employed |  |
| 11 | Prefer not to answer |  |
